# Clinical Report of Probable Catastrophic Antiphospholipid Syndrome in Pregnancy

**DOI:** 10.1155/2018/4176456

**Published:** 2018-04-03

**Authors:** Eryk Hakman, Sasha Mikhael

**Affiliations:** ^1^American University of the Caribbean, Coral Gables, FL, USA; ^2^Department of Obstetrics and Gynecology, Michigan State University College of Human Medicine, Providence Hospital, Southfield, MI, USA

## Abstract

**Background:**

Catastrophic APS (CAPS) is a rare but life-threatening form of APS defined as multiorgan thrombosis affecting a minimum of three organs with confirmation by histopathology of small vessel occlusions in at least one organ or tissue. The development of CAPS in pregnancy poses many diagnostic challenges as a result of its broad range of clinical presentations and its overlap with other obstetric complications and microangiopathic diseases. Because of the high associated mortality rate, prompt recognition and treatment are paramount.

**Case:**

A twenty-five-year-old G3P0111 with a history of multiple thromboembolisms presented at 21 weeks and 3 days of gestation with complaints of right upper quadrant pain, visual disturbances, headache, and syncopal episodes. Laboratory evaluation demonstrated microangiopathic disease with hemolysis (confirmed on peripheral smear), elevated liver enzymes, and abnormal 24-hour urine protein with vital signs within the normal range. Presence of significantly elevated antiphospholipid antibodies was noted, facilitating the diagnosis of probable CAPS. Proper workup was achieved based on clinical suspicion, allowing immediate and appropriate management.

**Conclusion:**

CAPS is a life-threatening condition rarely seen in pregnancy making early recognition difficult. A low threshold to initiate urgent and aggressive treatment should be maintained to minimize the risk of adverse outcomes.

## 1. Introduction

Antiphospholipid syndrome (APS) is an autoimmune disorder resulting in a hypercoagulable state due to the involvement of anticardiolipin, lupus anticoagulant, and anti-beta-2-glycoprotein-1 autoantibodies. APS is associated with the development of arterial and venous thrombi, most commonly manifested as deep venous thrombosis and pulmonary embolism [1]. Catastrophic APS (CAPS) is a rare but life-threatening form of APS defined as multiorgan thrombosis, affecting a minimum of three organs with confirmation by histopathology of small vessel occlusion in at least one organ or tissue [2] and presence of antiphospholipid (aPL) antibodies on two separate occasions, six weeks apart [2, 3]. CAPS rapidly develops and has a reported mortality rate as high as 50% making its prompt diagnosis critical [4]. Maternal clinical manifestations are nonspecific and include abdominal pain, elevated liver enzymes, encephalopathy, altered mental status, seizure, stroke, chest pain, hypertension, proteinuria, renal insufficiency, dyspnea, and pulmonary embolism, to list a few. Additionally, fetal morbidities are observed as a result of placental insufficiency leading to growth restriction, preterm birth, or death [1]. The development of CAPS in pregnancy poses many diagnostic challenges as a result of its broad range of clinical presentations and its overlap with other obstetric complications and microangiopathic diseases. Because of the high associated mortality rate, rapid recognition and treatment are paramount. To help mitigate these challenges, we present a case of a twenty-five-year-old with probable CAPS in pregnancy.

## 2. Case

A twenty-five-year-old gravida 3 para 0111 had established limited prenatal care at our office at 9 weeks of gestation in conjunction with maternal fetal medicine for a known history of deep venous thrombosis (DVT) and pulmonary emboli (PE). The patient had multiple PEs and DVTs over the course of six years and was instructed to continue lifelong anticoagulation; however, the patient failed to maintain compliance until this pregnancy where she was placed on enoxaparin 100 mg twice daily. Her obstetrical history was significant for a preterm cesarean delivery at approximately 33 weeks of gestation, for arrest of dilation after failed induction of labor for preeclampsia two years before. This was followed by a missed abortion at 16 weeks of gestation requiring a dilation and extraction six months prior to the current gestation. She had no notable gynecologic or social history. Her family history included multiple family members with ischemic cerebrovascular accidents. The patient did not follow up again until 21 weeks and 3 days of gestation for a visit with maternal fetal medicine and was immediately sent to labor and delivery for prompt evaluation of her symptoms. This included headache, nausea, vomiting, syncopal episodes, right upper quadrant pain, and blurred vision. Her vital signs were stable and she remained normotensive; however, due to her history of preeclampsia, a 24-hour urine protein level was collected in addition to liver function panel, blood urea nitrogen, and creatinine for assessment of renal function. An EKG was also obtained due to complaints of syncopal episodes. Given her history of multiple PEs and DVTs, a thorough coagulopathy workup was done including antiphospholipid antibodies. Lab abnormalities included lupus anticoagulant antibody via Russell Viper Venom test in addition to anticardiolipin IgG titers > 112 and elevated IgM titers (74.5). The patient left against medical advice before completion of her workup and presented 10 days later to labor and delivery with complaints of worsening nausea, vomiting, chest pain, headache, and shortness of breath. On examination, vital signs remained stable including a blood pressure of 126/87 and pulse oximetry at 100% with her pulse at 56 beats per minute. She was noted to have significant right upper quadrant tenderness on superficial palpation. Her cervix was 0 cm dilated and 0% effaced, appropriate for gestational age. Fetal heart tones were noted to be 160 beats per minute. Lab evaluation revealed a platelet count rapidly declining within hours of presentation from 111,000/mcL to 58,000/mcL. SGOT and SGPT were noted to be 179 U/L and 194 U/L (from a baseline of 23 U/L and 21 U/L, resp.). EKG revealed normal sinus rhythm; additionally, troponins were negative; however lactate dehydrogenase remained as high as 480 U/L. Haptoglobin was also noted to be <10 mg/dL, well below the normal range suggestive of microangiopathic hemolytic anemia (MAHA), confirmed by peripheral smear. Anti-Xa was 0.7 IU/mL (within the therapeutic range) demonstrating patient compliance with anticoagulants. The remainder of her comprehensive lab workup was within normal limits. Due to significant and rapid decline in platelet count, conversely elevated liver function tests, and 24-hour urine protein of 329 mg, a preliminary diagnosis of HELLP was made and induction of labor was initiated. Given the patients' critical prognosis with rapidly worsening lab findings, immediate delivery via cesarean section was undertaken. A central line was placed prior to surgery and a Jackson-Pratt drain placed intraoperatively due to critical platelet levels. The patient was brought to the intensive care unit (ICU) postoperatively where she was continued on a magnesium drip since there was concern for HELLP. After minimal clinical improvement, hematology, critical care, and cardiology teams were consulted for a multidisciplinary approach. All teams reached a consensus for a diagnosis of probable CAPS and intravenous heparin infusion was initiated. Brain imaging was not performed since clinical improvement in neurologic manifestations drastically improved. Placental pathology reported focal fetal thrombotic vasculopathy characterized by microthrombi within villous vessels with decidual and adjacent placental infarct. She achieved clinical stability after postoperative day (POD) 2 and was transitioned to enoxaparin 100 mg twice daily. On POD 3 she was transferred out of the ICU and continued to meet all postoperative goals allowing for discharge in stable condition POD 4. The patient was instructed to follow-up in the office with plans to repeat lab workup for confirmation of APS.

## 3. Discussion

CAPS remains a diagnostic challenge as its broad range of clinical signs, symptoms, and laboratory findings, as seen in this case, often overlap with other obstetrical complications including HELLP syndrome, thrombotic thrombocytopenic purpura (TTP), and acute fatty liver of pregnancy (AFLP). Given the patient's nonspecific presentation including right upper quadrant pain, nausea, vomiting, headache, visual disturbances, elevated liver enzymes, low platelets, and haptoglobin, achieving a definitive diagnosis in this acute setting was involved. The above listed differentials were considered with particular focus on HELLP syndrome. HELLP is a common obstetrical complication that is observed with a triad of hemolysis (suggested by an abnormal peripheral smear, low haptoglobin, and elevated bilirubin), elevated liver enzymes with AST, and ALT twice the upper limit of normal and low platelets < 100,000/mcL. In this case however, based on the patient's history of multiple thromboembolic events and the presence of aPL antibodies, she was appropriately classified as having probable CAPS. Although the patient only had one laboratory confirmation for aPL antibodies (rather than two values, six weeks apart per diagnostic criteria), a high index of suspicion is required as CAPS results in rapid deterioration of the mother and fetus. With an established history of APS, CAPS can be more easily identified. Alternatively, in the setting of an unknown history of APS, recognition of CAPS proves to be more complex and if not part of the differential can easily be missed [2]. It is important for the clinician to be mindful that CAPS should be considered as being part of a spectrum including definite or probable CAPS and CAPS-like disease [5]. Diagnostic criteria for definite CAPS include microthrombi in three or more organs, development of symptoms in less than one week, histopathological confirmation of microthrombi in one organ, and the presence of aPL antibodies on two occasions that are at least six weeks apart [6]. If only three out of the four criteria are met, probable CAPS is presumed as proposed by Asherson et al. When patients are aPL positive but do not meet criteria for classification of probable CAPS, then CAPS-like disease is considered, requiring close monitoring for the development of overt CAPS and are thus managed similarly.

This patient met three of the four criteria. Her headache and visual disturbances, abnormal liver enzymes, and elevated 24-hour urine protein were suggestive of intracranial, liver, and renal involvement, respectively, thus meeting Asherson's criteria for the diagnosis of probable CAPS. Although brain imaging was not performed, neurologic deficiency was presumed from acute onset of visual disturbances and a headache that was not relieved by pharmacotherapy. This case highlights the importance of gathering a thorough history, as mortality rates have been cited to be as high as 50%. Current studies, however, suggest mortality rates to have decreased to approximately 30% due to recent efforts in better identifying and treating CAPS [6]. Unfortunately, our patient was noncompliant and lost to follow-up between 9 and 21 weeks of gestation, preventing proper APS workup. Despite not having a formal APS evaluation, or cerebral imaging to confirm intracranial thrombosis, treatment for CAPS should be implemented in a timely fashion. The consensus among authors has been to initiate treatment if clinical suspicion is considerable, despite not fulfilling all CAPS diagnostic criteria, as was done in our case [5].

Current treatment involves anticoagulation. Due to CAPS only occurring in 1% of patients with APS, recommendations are based on case reports and expert opinions [2]. Consensus data recommends that pregnant patients should be started on a low molecular weight heparin prophylaxis, dosed at 0.5 mg/kg/day or unfractionated heparin at 5000 units twice daily as soon as pregnancy occurs, along with 81 mg low dose aspirin [7]. Full anticoagulation with heparin dosed at 1 mg/kg every twelve hours is recommended if the patient had a prior history of thrombosis. Treatment with anticoagulation should persist for at least six weeks postpartum. Limited case reports and small studies describe the addition of prednisone, hydroxychloroquine, and intravenous immunoglobulin (IVIG) with favorable outcomes [8–11]. The initial diagnosis of HELLP syndrome, in this case, clouded the clinical suspicion for probable CAPS, resulting in a delay in appropriate treatment. Despite expeditious delivery and initiation of IV heparin resulting in rapid clinical improvement, corticosteroids should have been added to the patient's management to minimize morbidity. In contrast to other obstetrical complications such as preeclampsia, where definitive treatment is delivery, it remains uncertain if delivery treats CAPS. Regardless, current recommendations are to remove triggering factors, which includes pregnancy. Therefore, in theory, timely delivery should improve patient outcome and perhaps even be curative.

In conclusion, CAPS is a life-threatening condition rarely seen in pregnancy making early recognition difficult. A low threshold to initiate urgent and aggressive treatment should be maintained even with the slightest suspicion, regardless of whether confirmatory aPL testing was obtained. This may perhaps contribute to improved maternal and fetal morbidity and further lower mortality rates.

## Data Availability

The data used to support the findings of this study are available from the corresponding author upon request.
